# Hypoparathyroidism after Roux-en-Y gastric bypass - a challenge for clinical management: a case report

**DOI:** 10.1186/1752-1947-8-357

**Published:** 2014-10-28

**Authors:** Diogo Guarnieri Panazzolo, Tássia Gomide Braga, Anice Bergamim, Bárbara Pires, Hirlena Almeida, Luiz Guilherme Kraemer-Aguiar

**Affiliations:** 1 Obesity Unit, Polyclinic Piquet Carneiro; Endocrinology, Department of Internal Medicine, Medical Sciences Faculty, State University of Rio de Janeiro, Av. Marechal Rondon, 381, Rio de Janeiro 20950-003, Brazil

**Keywords:** Bariatric surgery, Hypocalcemia, Hyperparathyroidism

## Abstract

**Introduction:**

In this report, we describe challenges we encountered in the clinical management of a patient with hypoparathyroidism who had previously undergone a bariatric procedure.

**Case presentation:**

We report the case of a 38-year-old Caucasian woman who had undergone a Roux-en-Y gastric bypass procedure for treatment of obesity. She also had a past history of right lobectomy to treat a benign thyroid nodule. Another thyroid nodule was diagnosed after her bariatric surgery, so a new thyroid surgery was performed. Permanent hypoparathyroidism occurred after the second thyroid surgery. A Roux-en-Y gastric bypass resulted in important weight loss, but the preferential site of calcium absorption was bypassed. The lack of endogenous parathyroid hormone secretion due to post-surgical hypoparathyroidism abolished the physiological mechanism that compensates the reduced calcium absorption, which was a challenge for us to overcome. In this report, we describe our clinical therapeutic choices to maintain normocalcemia and normophosphatemia in this patient. Higher doses of exogenous calcium citrate, calcitriol and cholecalciferol were used, but hypocalcemia was still present. To improve vitamin D absorption with resultant improvement of calcium homeostasis, we speculated that adding pancrelipase to meals would increase lipid absorption and possibly fat-soluble vitamins, including vitamin D. Only after the addition of pancrelipase did the patient improve without weight regain according to clinical and laboratory assessments.

**Conclusion:**

The use of exogenous pancreatic enzymes improved calcium homeostasis in this bariatric patient. The role of these enzymes on vitamin D absorption and subsequent rise in calcium levels in hypoparathyroid patients who undergo bariatric procedures need further investigation.

## Introduction

Obesity affects over 300 million individuals worldwide, and many of them are surgically treated. Roux-en-Y gastric bypass (RYGB) is a bariatric procedure aimed at weight reduction in morbidly obese patients. It bypasses the preferential sites for calcium and vitamin D absorption, placing patients at risk for altered calcium homeostasis. To compensate the secondary hypocalcemia, bariatric patients are prone to augment parathyroid hormone (PTH) levels.

Thyroid nodules are detected by ultrasound in 19% to 67% of randomly selected individuals. Once detected, thyroid cancer should be excluded. A relationship between excessive adiposity and thyroid cancer risk has been established [1,2], which puts obese as well as bariatric patients at risk for undergoing thyroidectomy. Primary hypoparathyroidism, one of the most serious complications following thyroid surgery, is characterized by hypocalcemia and hyperphosphatemia with absent or inappropriately low levels of PTH. Intestinal absorption of calcium and vitamin D is fundamental in the clinical management of hypoparathyroidism.

In this case report, we describe a bariatric patient who underwent total thyroidectomy for a thyroid nodule that was complicated with permanent hypoparathyroidism. Our goal is to emphasize the difficulties in the clinical management of hypoparathyroidism in a bariatric patient and to relate our therapeutic choices to achieve normocalcemia.

## Case presentation

A 38-year-old Caucasian woman was diagnosed with a left-sided neck nodule six months after undergoing RYGB to treat obesity. She recalled palpating a vague mass on the cervical site before her RYGB, but this mass became more pronounced only after she lost 33kg. (Her body mass index (BMI) had changed from 43.5kg/m^2^ to 30.5kg/m^2^). She reported a past history (prior to her RYGB) of a right lobectomy of the thyroid to treat a benign nodule, as well as hypertension and obstructive sleep apnea. Before undergoing the second thyroid surgery and during her first visit at our clinic, she brought laboratory results showing normal levels of calcium, phosphorus, 25-hydroxyvitamin D (25(OH)D), PTH and thyroid-stimulating hormone (TSH).

To investigate the cervical mass, a thyroid ultrasound was performed. The scan showed a 3.1×2.2×2.0cm hypoechoic nodule on the left lobe. Subsequently an aspirative biopsy was obtained. Cytopathology suggested a Hurthle cell lesion. Total thyroidectomy was indicated and hence carried out, and the diagnosis of Hurthle cell adenoma was confirmed. Immediately after surgery, the patient had hypocalcemia and required an intravenous calcium gluconate infusion to stabilize her calcemia. All of the laboratory data obtained during clinical management are provided in Table 1.

**Table 1:** **Laboratory variables during follow-up**^**a**^

	**Total calcium (mg/dl)**	**Phosphorus (mg/dl)**	**25(OH)D (μg/L)**	**PTH (pg/ml)**	**Drugs used to manage calcium metabolism (dose/day)**
Normal ranges	8.4 to 10.2	3.5 to 5.0	30 to 80	10 to 65	
After RYGB and before TT	9.0	4.2	64.4	27.9	
2 months post-TT	5.9^b^	7.1	–	–	Calcium carbonate (7.5g)
					Calcitriol (2μg)
3 months post-TT	6.2^b^	6.8	76.6	7.1	Calcium carbonate
					Calcitriol (2μg)
4 months post-TT	6.8^b^	6.8	16.4	–	Calcium citrate (12g)
					Cholecalciferol (12,000IU)
					Calcitriol (2μg)
6 months post-TT	7.2^b^	5.0	–	–	Calcium citrate (12g)
					Cholecalciferol (12,000IU)
					Calcitriol (2μg)
					Pancrelipase (3 capsules/day)
8 months post-TT	7.8^b^	5.2	67.9	8.0	Calcium citrate (14g)
					Cholecalciferol (12,000IU)
					Calcitriol (2μg)
					Pancrelipase (3 capsules/day)
14 months post-TT, pregnant second trimester	7.1^b^	5.2	49.2	5.9	Calcium citrate (14g)
					Cholecalciferol (12,000IU)
					Calcitriol (1.5μg)
					Pancrelipase (3 capsules/day)
Last measurements	8.8^b^	5.1	34.8	–	Calcium citrate (18g)
					Cholecalciferol (12,000IU)
					Calcitriol (1.5μg)
					Pancrelipase (3 capsules/day)

^a^25(OH)D, 25-hydroxyvitamin D; PTH, Parathyroid hormone; RYGB, Roux-en-Y gastric bypass; TT, Total thyroidectomy. ^b^Total calcium was corrected by albumin levels.

Two months after her total thyroidectomy, she was referred to our unit’s care center by the surgeons because of difficulties in the clinical management of hypoparathyroidism. Meanwhile, she was admitted several times to an emergency room to reverse hypocalcemia with intravenous calcium infusions. During her visit at our unit, she reported paresthesias, muscle cramps and tingling in the mouth. A physical examination revealed that she had positive Chvostek’s and Trousseau’s signs. Her serum calcium and phosphorus levels were 5.9mg/dl and 7.1mg/dl, respectively. Her TSH level was 16.6μUI/ml. She was being treated with levothyroxine 100μg/day, calcium carbonate 7.5g/day, oral calcitriol 2.0μg twice daily and a multivitamin tablet. Doses of levothyroxine and calcium carbonate were increased to 150μg/day and 12g/day, respectively, and magnesium oxide 400mg tablet three times per day was added. Strict instructions about sunlight exposure (20min/day) and diet were given. All these measures improved her calcium homeostasis and avoided any further hospitalization. At her three-month follow-up examination, it was discovered that her calcium level had increased and her phosphorus level had decreased (see Table 1). She had a low PTH level and a normal TSH level. Her magnesium and albumin levels and renal and hepatic function were within normal ranges (NRs). We decided to change calcium carbonate to citrate (12g/day) and to add cholecalciferol (12,000IU/day).

One month later, her symptoms were partially improved, and she reported that our advice about sunlight exposure was not being completely followed. Minimal changes in laboratory data were noticed, and her urinary calcium/creatinine ratio was 0.09 (NR<0.20). Because of the large amount of tablets/pills needed for daily intake at that time, we speculated that she could be a candidate for surgical reversal of the bariatric procedure; however, she definitively rejected it, fearing weight regain. Facing this challenge, and without financial resources to cope with synthetic PTH hormone treatment, we hypothesized that the use of pancrelipase could improve fat absorption at the alimentary tract with possible positive effects on vitamin D absorption and subsequently on calcium homeostasis. To test our hypothesis, pancrelipase was added at mealtime (three times per day) (compound capsule containing 10,000 USP units of lipase, 37,500 USP units of protease and 33,200 USP units of amylase). After continuous use of pancrelipase without side effects, her symptoms gradually improved until she became totally asymptomatic. Two months later, she had serum calcium and phosphorus levels of 7.2mg/dl and 5.0mg/dl, respectively. We increased her calcium citrate dosage to 14g/day. After two months, her levels of calcium, phosphorus and 25(OH)D were 7.8mg/dl, 5.2mg/dl and 67.9μg/L, respectively. At that time, her calciuria was 62.6mg/24h (NR, 50mg/24h to 250mg/24h), and her phosphaturia was 405mg/24h (NR, 340mg/24h to 1300mg/24h). Calcitriol was subsequently reduced to 1.5μg three times daily.

Her symptoms had completely remitted at 10 months after thyroidectomy. Her levels of calcium were within, and eventually slightly below, the NR. She became pregnant, and, during the second trimester, although asymptomatic, she had hypocalcemia with normal levels of phosphorus and 25(OH)D (Table 1, 14 months). Calcium citrate was increased to 18g/day, which stabilized calcium homeostasis from the sixth month of pregnancy until delivery. She gave birth to a healthy newborn at term and breastfed her baby during the first six months. At present, her symptoms have remitted and her calcium, phosphorus and 25(OH)D levels are within NR. However, she still ingests an excessive daily number of tablets and/or pills (a total of 45/day). She has not regained weight (65kg; BMI, 25.7kg/m^2^).

## Discussion

After bariatric surgery, and independently of multivitamin and/or mineral supplementation, the incidence of hypocalcemia ranges from 15% to 48% and vitamin D deficiency ranges from 50% to 63% [3]. The duodenum and jejunum are the preferential sites for calcium absorption, and, after a RYGB, they are bypassed. The duodenum can absorb 80% to 100% of calcium by vitamin D–dependent, transcellular active transport, but, when it is bypassed, the calcium absorption takes place through a less efficient paracellular mechanism [4]. It is known that malabsorption of fat-soluble vitamins caused by poor mixing of bile salts decreases the amount of vitamin D available and contributes further to deficient calcium homeostasis [3]. Additionally, partial gastrectomy reduces gastric acidity, resulting in an impaired absorption of calcium salts. Complicating this scenario is that these patients usually follow a low-calcium diet because of low tolerance of calcium-rich dairy products [4]. Therefore, to stabilize calcium levels, secondary hyperparathyroidism may occur. However, in the present case, the absence of functional parathyroids precluded the physiological compensatory mechanism. Therefore, we focused treatment on improvement of the intestinal absorption of calcium. Considering that it is well established that cutaneous synthesis of vitamin D is responsible for up to 90% of its source, we first recommended increased calcium dietary intake and sunlight exposure. Calcium carbonate was changed to calcium citrate to improve calcium absorption, which was impaired by achloridia after RYGB [4]. Pancreatic exocrine insufficiency is a possible complication of partial or total gastrectomy [5,6]. Originally, in an attempt to improve intestinal calcium absorption, we added pancrelipase to meals, based on speculation that lipids, and consequently fat-soluble vitamins (specifically vitamin D), could have an improved absorption with possible beneficial effects on calcium homeostasis. The indication for substitutive therapy with pancreatic enzymes in asymptomatic patients is debatable. However, a study has demonstrated that patients with asymptomatic steatorrhea and consistently low levels of vitamins (such as liposoluble vitamins) can revert to normal status with pancreatic enzyme substitution therapy [7]. Fortunately, associated with other measures that we employed, this therapeutic choice produced successful results based on clinical and laboratory test results.

Other therapeutic measures, such as thiazides, to enhance distal renal tubular calcium reabsorption are used as adjunctive therapy for hypoparathyroidism [8]. In the present case, however, because of low systolic blood pressure (90mmHg) and calciuria, we did not recommend such therapy. Daily costs of synthetic PTH therapy prevented an indication for this therapy, although it is known that in hypoparathyroidism it is able to reduce daily requirements of calcium and vitamin D, of calciuria and of ectopic soft-tissue calcification, while improving bone health [9].

Magnesium has a critical role as an activator of many ionic carriers, which is essential not solely for neuromuscular excitability but also for transcription of the PTH gene and for binding of PTH to type 1 receptors in the bones and kidneys [10]. As we did not know whether the patient would have an intact parathyroid, we prescribed magnesium supplementation primarily to improve neuromuscular hyperexcitability symptoms. Despite the fact that magnesium deficiency is relatively rare in a bariatric patient, it can occur after RYGB with severe and refractory symptomatic hypocalcemia.

A few studies in which clinicians followed bariatric patients with hypoparathyroidism have demonstrated the usefulness of concomitant replacement of calcitriol and cholecalciferol in improving calcium levels [11,12]. Calcitriol may improve hypocalcemic symptoms faster because it has a biological half-life of four to six hours, whereas cholecalciferol requires hydroxylation in the liver and kidneys to become active and may take two to three weeks to produce its effects.

Clinical and laboratory improvement was observed before our patient’s pregnancy. However, there is some evidence that this condition is related to stabilization of calcium levels because the placenta is an external source of calcitriol [13]. Additionally, lactation is associated with increased serum levels of PTH-related protein and alkaline phosphatase, which results in a rise in calcemia [14]. These two physiological conditions might have aided our patient’s calcium homeostasis during pregnancy and lactation.

Although recently our patient’s hypoparathyroidism has been well compensated, her quality of life is somewhat impaired by the high number of tablets and pills that must be ingested, and she is still at risk for hypocalcemia. Reversal of RYGB due to refractory hypoparathyroidism remains a possible option for this patient in long-term follow-up [15].

## Conclusions

This case illustrates the challenge for the clinical management of hypoparathyroidism in a bariatric patient. The value of exogenous pancreatic enzymes on calcium and vitamin D absorption remains speculative and needs further investigation.

## Consent

Written informed consent was obtained from the patient for publication of this case report and any accompanying images. A copy of the written consent is available for review by the Editor-in-Chief of this journal.

## Abbreviations

25(OH)D: 25-hydroxyvitamin D; BMI: Body mass index; NR: Normal range; PTH: Parathyroid hormone; RYGB: Roux-en-Y gastric bypass; TSH: Thyroid-stimulating hormone; USP: United States Pharmacopoeia.

## Competing interests

The authors declare that they have no competing interests.

## Authors’ contributions

DGP conceived of and designed the report, collected and analyzed data and drafted the manuscript. TGB collected and analyzed data and drafted the manuscript. AB, BP and HA collected and analyzed data. LGKA conceived of and designed the report, drafted the manuscript and revised it critically. All authors read and approved the final manuscript.
